# Inhibitory Effects of Aquadag, a Black Carbon Surrogate, on Microbial Growth via Surface-Mediated Stress: Evidence from Adenosine Triphosphate Assay

**DOI:** 10.3390/toxics13090719

**Published:** 2025-08-27

**Authors:** Hwangyu Yoo, Saehee Lim, I Seul Cho, Haneul Im, Euna Lee, Siyoung Choi, Han-Suk Kim, Sohee Jeong, Younggyun Choi

**Affiliations:** 1 Department of Environmental Engineering, Chungnam National University, Daejeon 34134, Republic of Korea; hwankuy97@gmail.com (H.Y.); youngchoi@cnu.ac.kr (Y.C.); 2 Department of Environmental IT Convergence Engineering, Chungnam National University, Daejeon 34134, Republic of Korea; iseuljo22@gmail.com (I.S.C.); dlagksmf3020@gmail.com (H.I.); pringles6946@gmail.com (E.L.); swing0128@gmail.com (S.C.); sosohee9@naver.com (S.J.); 3 Department of Earth and Environmental Sciences, Korea University, Seoul 02841, Republic of Korea; hskim77@dm-ent.co.kr; 4 R&D Center, Dong-Myung Enterprise, Seoul 06725, Republic of Korea

**Keywords:** particulate matter, black carbon, microbial growth, adenosine triphosphate (ATP), human health

## Abstract

Black carbon (BC) from incomplete combustion sources including traffic emissions affects human health due to its physical characteristics and ubiquity in urban environments. We examined the effects of BC on microbial growth in the presence of particulate matter (PM), using Aquadag as a surrogate for BC. Brunauer–Emmett–Teller analysis showed BC had a specific surface area of 123.2 m^2^ g^−1^, with over 90% of particles smaller than 100 nm, indicating strong surface interaction potential. Pseudomonas aeruginosa PA14 was cultured for 7 days with various BC concentrations and fixed PM. Increasing BC (0–100 ng mL^−1^) significantly inhibited growth, evidenced by a decline in cellular adenosine triphosphate (cATP) with a slope of −1.296 ± 0.258 cATP ng mL^−1^/BC ng mL^−1^. The seven-day mean cATP slope ranged from 77 to 131, with control at 161. The biomass stress index (BSI) increased by 56%, rising from 28.6 ± 8.8% (control) to 44.6 ± 16.1% under high BC. The BSI change was minimal on day 1 (<+0.1% per BC ng mL^−1^) but greater on days 5 (+0.125 ± 0.052%) and 7 (+0.130 ± 0.075%). BC does not cause immediate microbial death, but prolonged exposure induces cumulative stress, damages synthetic enzymes, inhibits growth, and may lead to cell death, with potential public health implications.

## 1. Introduction

Atmospheric particulate matter (PM) significantly impacts air quality, climate systems, and public health. Of particular concern is fine particulate matter (PM_2.5_), which refers to particles with aerodynamic diameters less than or equal to 2.5 μm. These particles consist of a complex mixture of substances either emitted directly into the atmosphere from both anthropogenic and natural sources (primary) or formed through chemical reactions in the atmosphere (secondary). PM_2.5_ can carry substantial amounts of toxic materials, penetrate deep into the lungs, and enter the bloodstream, potentially causing numerous serious health problems. These include respiratory disease [1,2], cardiovascular diseases [3,4], neurological diseases [5,6], cancer [7,8,9,10], and metabolic disease [11]. Despite growing evidence of adverse health impacts related to PM_2.5_ exposure, comprehensive data on long-term effects and associated morbidity remain limited in many countries.

Black carbon (BC) is a carbonaceous component of PM_2.5_, generated from the incomplete combustion of fossil fuels and biomass. In urban areas, its primary source is emissions from traffic-related activities [12]. As a prominent light-absorbing aerosol, BC strongly absorbs radiation within the visible spectrum [13]. Its warming effect influences atmospheric vertical stability and alters cloud distribution, thereby affecting the overall radiation balance [14]. In addition to its environmental and climatic impacts, BC exposure is associated with notable health risks. Epidemiological studies have shown significant links between BC and cardiovascular diseases [15,16], as well as respiratory disease [17,18]. Once inhaled, ambient BC can reach the human brain, potentially contributing to neurodegenerative disease development [19]. It was further demonstrated that BC particles can pass through the placental barrier and accumulate in fetal organs, raising serious concerns about prenatal vulnerability to air pollution [20]. Furthermore, recent cohort studies have reported that among PM_2.5_ components, BC shows the strongest association with all-cause mortality risk [21,22].

In recent years, a limited number of experiments have reported that exposure of pathogens to BC induces structural, compositional, and functional changes in biofilms, most notably biofilm resistance to multiple antibiotics and proteolysis [23]. Exposure to ultrafine BC decreases mitochondrial membrane potential and induces overproduction of reactive oxygen species (ROS), which are crucial for ATP synthesis and redox regulation [24]. This oxidative stress activates mitophagy to remove the damaged mitochondria; however, if the damage is excessive, accumulated mitochondrial loss can lead to energy depletion and ultimately trigger cell death [25]. In addition, continuous exposure to low-dose BC may induce a suppression in metabolic activity and cellular senescence in various lung-derived cell lines, including epithelial cells and macrophages. This indicates that even sub-cytotoxic levels of BC can impair cellular vitality and promote cellular aging [26]. Although growing evidence suggests the potential toxicity of BC, its biological impact on microbial growth and energy metabolism remains poorly understood, especially when assessed with direct physiological indicators of microbial energy capacity and viability.

Adenosine triphosphate (ATP) is a crucial molecule for cellular energy transport and processes. ATP bioluminescence has become a reliable method for detecting bioaerosols, unaffected by non-bioaerosols [27], and for evaluating platforms measuring microbial populations in air [28,29,30]. The ATP assay measures ATP to assess cell activity and viability [31], serving as a proxy for total viable biomass due to its correlation with organic carbon and intact cell counts [32,33,34]. Its simplicity and rapid assay time have driven its use [31].

This study aims to evaluate the effects of BC on microbial growth by monitoring Pseudomonas aeruginosa PA14 under prolonged exposure to PM and BC. While previous studies have primarily relied on indirect microbial indicators such as biofilm morphology, mitochondrial membrane potential, and reactive oxygen species (ROS) generation, we directly quantified bacterial metabolic activity using ATP bioluminescence as a proxy for microbial activity, enabling time-resolved assessment of cellular energy capacity. Generating a 7-day quantitative profile of ATP production across multiple BC exposure levels and using it to assess metabolic activity provides unprecedented, time-resolved experimental evidence of the impact of BC on PA14, offering new insights into pollutant–pathogen interactions at the metabolic level.

## 2. Materials and Methods

### 2.1. Materials

#### 2.1.1. Particulate Matter

The PM used in this study was a certified standard material (KRISS CRM 109-02-004) provided by the Korea Research Institute of Standards and Science (KRISS), containing known fractions of elements and PAHs resembling urban PM (Table 1). The material was prepared from PM collected over two years (2019–2021) from the intake filters of large buildings in Seoul and surrounding areas. Particles smaller than 20 µm were recovered by sieving, yielding 2.5 kg of homogenized PM from 6.2 kg of raw material. The homogenized material was stored in argon-filled 10 mL brown glass bottles, each containing at least 2 g, and sealed in aluminum-lined plastic bags. Detailed elemental composition and mass fraction, along with PAH analysis results, are presented in Table 1. Elemental concentrations were further quantified using inductively coupled plasma–mass spectrometry (ICP/MS) and wavelength-dispersive X-ray fluorescence spectroscopy (WD-XRF) at KRISS (Table 2).

#### 2.1.2. Aquadag—BC Reference Material

Aquadag (Acheson Inc., Port Huron, MI, USA), a colloidal dispersion of irregular graphite flakes suspended in approximately 80% water [36,37], was used in this experiment as a standard for BC. Aquadag has been widely applied in the calibration of instruments for atmospheric BC observations due to its known effective density being similar to ambient BC [38,39,40]. According to the product data sheet, Aquadag has a bulk density of 1.12 kg L^−1^. 

The morphology of ambient BC is typically small spherical aggregates with a size range between 20 and 50 nm, which is expected to facilitate effective surface reactions. The small size and significant porosity of BC particles may play an important role in surface interactions with microorganisms. The porosity and size distribution of Aquadag were analyzed using the Brunauer–Emmett–Teller (BET) method and a single-particle soot photometer (SP2; Droplet Measurement Technologies, Longmont, CO 80503, USA) coupled with an atomizer, respectively. BET measurements are an experimental method for determining the surface area of porous materials, in which a specific gas is adsorbed onto a sample at a constant temperature, and the amount of adsorbed gas is measured for calculating the surface area of the sample. The SP2 uses laser-induced incandescence to measure the mass of individual BC particles with diameters ranging between approximately 80 and 550 nm [41]. The particle number size distribution of BC atomized from Aquadag dispersion in deionized water was measured using the SP2.

#### 2.1.3. Bacterial Strains and Culture

Pseudomonas aeruginosa PA14 is a facultative anaerobic pathogenic bacterium with a broad host range, including humans, and is widely used as a model organism to study antibiotic resistance mechanisms and associated biological changes during infection [42,43]. The PA14 strain used in this study was supplied by Macrogen (Seoul, Republic of Korea). PA14 was cultured on Petri dishes at 30 °C for 24 h, and colonies were inoculated into AB medium and grown at 200 rpm. AB medium is a defined essential medium widely used for precise physiological studies of various microbial species, including Pseudomonas aeruginosa [44,45,46]. For cultivation, *P. aeruginosa* PA14 was grown on Luria–Bertani (LB) medium (Miller formulation; product code 244520; Difco™, BD, Franklin Lakes, NJ, USA) containing tryptone (10.0 g L^−1^), yeast extract (5.0 g L^−1^), sodium chloride (10.0 g L^−1^), and agar (15.0 g L^−1^). For liquid culture, cells were incubated in LB broth at 37 °C with shaking at 200 rpm using a shaking incubator (SI-150, Dongjin Science, Seoul, Republic of Korea). Overnight cultures of the bacterial strains were seeded into fresh Autoinducer Bioassay (AB) medium as follows. In this study, the AB medium was composed of NaCl (0.3 M), MgSO_4_·7H_2_O (0.05 M), and vitamin-free casamino acid (0.2%, DifcoTM, Thermo Fisher Scientific, Waltham, MA, USA) and adjusted to pH 7.5 with KOH (1 M) [47]. The total volume was made up to 970 mL using distilled water. Additions included potassium phosphate buffer (1 M, pH 7.0), L-arginine (0.1 M), and glycerol, with sterilization by autoclaving (15 min, 121 °C). LB broth containing 1.5% (*w*/*v*) agar was used to prepare plates [48]. Liquid media were prepared by sterilizing in an autoclave, and solid media were prepared by incorporating 1.5% agar into the liquid media and incubating the vials in a Petri dish on a rack at 30 °C for 2 days without shaking. For each 10 mL of AB medium, 1 mL of PA14 was placed in a tube, and the suspension was centrifuged at 12,000 rpm, resuspended, and added to AB medium. All medium components were purchased from Difco™ (BD, Franklin Lakes, NJ, USA), and the chemicals were purchased from Sigma-Aldrich, St. Louis, MO, USA.

### 2.2. Experiments

#### 2.2.1. Cultivation Setup

To better simulate atmospheric exposure in urban environments, experimental groups were prepared in which PA14 cultures were combined with PM and varying concentrations of Aquadag.

PA14 was cultured in 10 glass vials per set, each containing PA14 mixed with a solution of urban PM and Aquadag. Since PM and Aquadag often coexist in urban air as complex mixtures, this study maintained a fixed concentration of PM across all experimental conditions to simulate realistic exposure scenarios, with only the Aquadag levels being varied. The Aquadag stock solution (25,000 ng mL^−1^) was diluted to 1000 and 100 ng mL^−1^ to make working solutions. From these, we prepared eight concentration levels (ranging from 5 to 100 ng mL^−1^) of BC (Table 3). Triplicate cultures were used for each set of 10 vials to ensure the reliability and reproducibility of the data. In this study, cultures were incubated at 30 °C with 90 rpm, as temperature affects the virulence of pathogens [49], and some virulence-related pathways are not activated at temperatures below 30 °C [50].

#### 2.2.2. Measurement of ATP Levels

Based on the bioluminescence reaction in which the enzyme luciferase catalyzes a reaction using ATP and the substrate luciferin to produce light, the intensity of the emitted light is directly proportional to the concentration of ATP, allowing a quantitative determination of ATP in the sample [51,52,53]. We used a Quench-Gone Aqueous Test Kit (LuminUltra, Fredericton, NB, Canada) in conjunction with PhotonMaster (LuminUltra) to measure the total ATP (tATP), cellular ATP (cATP) and dissolved ATP (dATP) [49]. tATP reflects the overall energy status of the cell, including all forms of ATP in the cell. cATP is the ATP in living cells and reflects cellular metabolic activity, while dATP is the ATP remaining after cell death and is related to the number of dead cells. Subtracting dATP from tATP indicates the level of cATP (Equation (1)). This calculation has been widely applied in microbial activity monitoring, including in studies such as Liu (2019) [54].

(1) cATP=tATP−dATP

(2) $$BSI\left(\%\right)=\left(\frac{dATP}{tATP}\right)\times 100\;$$

**Figure 1:**
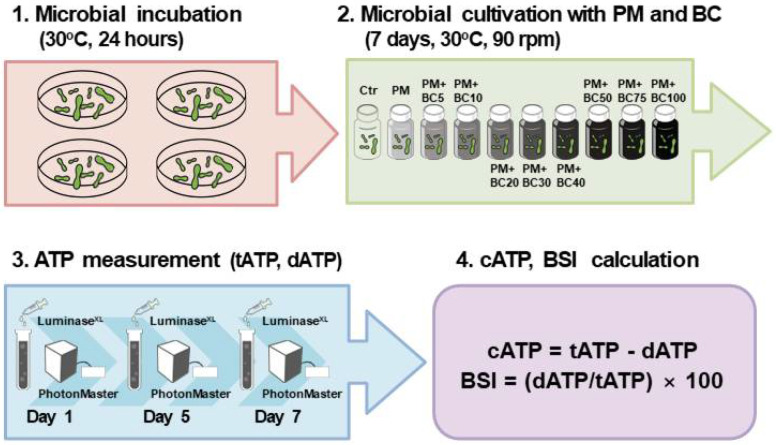
Schematic diagram of the experimental setup and procedure.

After measuring ATP, the biomass stress index (BSI) was calculated (Equation (2)), which has been employed as an indicator of microbial stress levels in recent research [55]. BSI indicates the stress level in an organism and represents the percentage of ATP in dead or stressed cells. It is predictable depending on the biomass and environmental conditions. Using these characteristics, this set of experiments quantitatively analyzed the degree of external pressure during microbial growth. ATP assays were performed on the first, fifth, and seventh days of incubation, and the relationship between BC and microbial growth was derived mainly from the changes in cATP and BSI values (Figure 1).

## 3. Results and Discussion

### 3.1. Surface and Size Characteristics of the Black Carbon Reference Material

**Figure 2:**
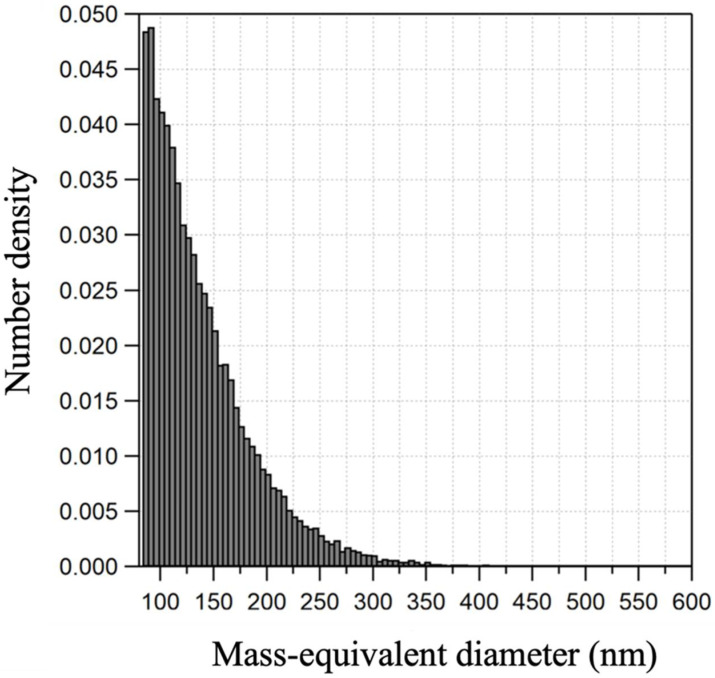
Number size distribution of Aquadag, a BC surrogate, obtained from SP2 measurements. Aquadag particles’ size is expressed in mass-equivalent diameter (see Section 3.1).

The BET analysis showed that the specific surface area of Aquadag was 123.2 m^2^ g^−1^. This was much larger than the specific surface area of commercially available aerosol standards (2.0–35.7 m^2^ g^−1^ [56]). The specific surface area of various BC particles widely used in industry is in the range between 30 and 160 m^2^ g^−1^ [57,58,59]. Figure 2 shows the number density of Aquadag particles as a function of particle size, where the mass-equivalent diameter was calculated assuming spherical, void-free particles with a density of 1.8 g cm^−3^. The particle number size distribution of Aquadag revealed that the particles were concentrated in the sub-100 nm diameter range. This similarity in particle size is aligned with observations from urban Seoul, where BC in ambient air is typically found to be smaller than 100 nm [60], underscoring the physical comparability between Aquadag and atmospheric BC. For this reason, Aquadag was selected and used in this study as a representative material for BC particles.

Aquadag’s surface area is higher compared to other materials, likely due to nanoparticle agglomeration (Figure 2). This property increases physical contact with microbial cells by providing a larger surface area. Previous studies have indicated a correlation between nanoscale particles, such as Aquadag, and increased cellular damage in microbial models [61], suggesting that surface area may be a more significant driver of toxicity than particle mass alone.

### 3.2. Microbial Growth Under the Presence of PM and BC

**Figure 3:**
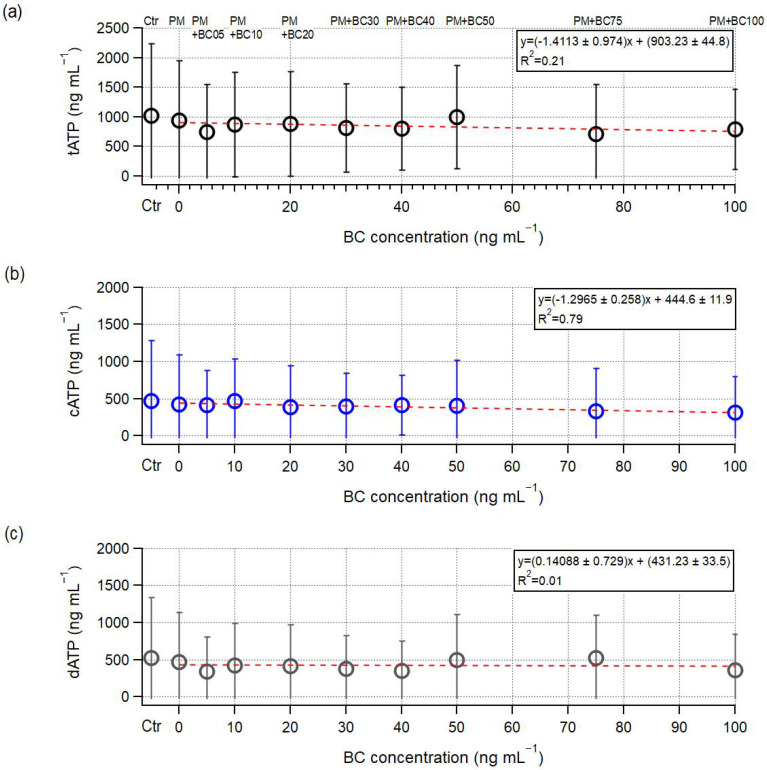
Variations in tATP, cATP, and dATP in incubated samples with different BC (Aquadag) concentrations: (**a**) tATP, (**b**) cATP, and (**c**) dATP. Each point with error bars denotes the mean ± standard deviation under different conditions (Ctr, PM, and PM + BC5–BC100). The red dashed line indicates the fitted trend, illustrating the influence of BC concentration on ATP dynamics.

To assess the concentration-dependent effects of BC on microbial activity, ATP levels were measured at three time points (days 1, 5, and 7) during the 7-day incubation period. Their average was used to represent the cumulative physiological response, reducing temporal variability and enabling comparison across BC levels. Figure 3 shows the mean levels of tATP, cATP, and dATP over the 7-day incubation period in each sample. In the sample containing PM alone (PM + BC0), the cATP levels were reduced compared with those of the control (Ctr), and this reduction became more pronounced as BC concentrations increased (slope = −1.296 ± 0.258 ng mL^−1^ cATP/ng mL^−1^ BC, R^2^ = 0.79, Figure 3b). Conversely, no clear trend was observed in dATP levels with increasing BC concentrations (R^2^ = 0.01, Figure 3c), suggesting that elevated BC exposure did not induce substantial microbial cell lysis. As a result, the tATP levels showed an overall decreasing trend with a slope of −1.411 ± 0.974 ng mL^−1^ cATP/ng mL^−1^ BC and R^2^ = 0.21 (Figure 3c). These results indicate that elevated BC concentrations primarily suppress microbial growth rather than causing cell death. This inhibitory effect may be associated with non-destructive interactions of carbon-based particles that alter microbial physiology without leading to cell death. These mechanisms may include physical disruption of the cell membrane structure, as reported in [62], where carbon nanomaterials caused moderate membrane damage in *P. aeruginosa* PG201 (albeit without apparent inhibition of bacterial growth). Additional mechanisms may involve suppression of metabolic activity [26] and oxidative stress-induced ATP reduction accompanied by elevated expression of bacterial stress response genes [63].

Table S1 provides detailed changes in cATP, dATP, and tATP levels according to BC concentration and exposure duration. The mean ± standard deviation values of cATP for all samples were 124.6 ± 42.8, 402.9 ± 112.9, and 816.0 ± 182.7 ng mL^−1^ on days 1, 5, and 7, respectively. In the Ctr samples, cATP levels increased from 80.7 ± 64.0 ng mL^−1^ on day 1 to 431.3 ± 72.4 ng mL^−1^ on day 5 and 1119.7 ± 151.9 ng mL^−1^ on day 7. The PM + BC0 samples showed a similar trend, with cATP levels of 96.1 ± 21.8, 416.6 ± 52.9, and 933.5 ± 239.6 ng mL^−1^ on days 1, 5, and 7, respectively. Until day 5, the cATP values of the PM + BC0 sample were within the margin of error of the Ctr. However, by day 7, the cATP level of PM + BC0 was approximately 17% lower than that of the Ctr. This result suggests that PM alone did not significantly suppress microbial activity in the early phase of exposure but may have begun to exert inhibitory effects over longer durations. This trend is further supported by the calculated slopes of cATP increase over the 7-day period. The average rate of cATP increase was 161 ng mL^−1^/7 days for the Ctr and 131 ng mL^−1^/7 days for the PM + BC0 sample. In the PM + BC-treated sample, the growth rate declined even more, with slopes ranging from 77 to 131 ng mL^−1^/7 days depending on BC concentration. These results highlight that although microbial growth occurred in all samples, the overall growth rate was reduced in the presence of PM and was further inhibited as BC concentration increased.

**Figure 4:**
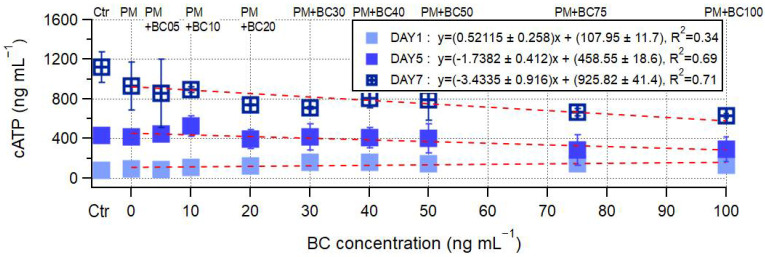
Changes in cATP over time (day 1, day 5, and day 7) in samples with varying BC (Aquadag) concentrations. The red dashed lines represent the trend lines of cATP changes.

In Figure 3b, the mean cATP levels across samples show an overall increasing trend with exposure duration; however, the magnitude of this increase varied substantially from sample to sample. On day 1, cATP levels varied between 96.1 ng mL^−1^ and 262.0 ng mL^−1^, a difference of 165.9 ng mL^−1^. This gap increased to 242.6 ng mL^−1^ on day 5 (ranging from 285.5 to 528.1 ng mL^−1^) and further to 304.2 ng mL^−1^ on day 7 (ranging from 629.3 to 933.5 ng mL^−1^). These expanding ranges of cATP reflect an increasing sample-to-sample variability in microbial response with prolonged exposure. Notably, the rank orders of samples based on cATP level on day 1 were not maintained on day 7, indicating that early metabolic responses did not consistently predict long-term outcomes. Despite this variability, a consistent pattern emerged with increasing BC concentration. As illustrated in Figure 4, cATP levels showed a clear negative correlation with BC concentration, particularly on days 5 and 7. The regression slope steepened over time, from −1.738 ± 0.412 ng mL^−1^/BC on day 5 (R^2^ = 0.69) to −3.434 ± 0.916 ng mL^−1^/BC on day 7 (R^2^ = 0.71), highlighting that the inhibitory effect of BC on microbial metabolic activity intensified with prolonged exposure.

**Figure 5:**
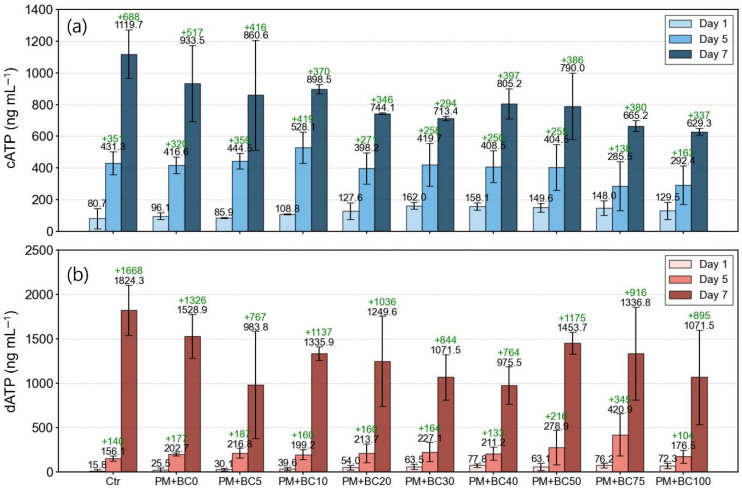
Changes in cATP (**a**) and dATP (**b**) over time (day 1, day 5, and day 7) across all samples. Black text above each bar indicates the measured average ATP concentration (ng mL^−1^) for each day. Green text represents the incremental change compared to the previous time point (i.e., the difference in cATP or dATP between day 5 and day 1 and between day 7 and day 5).

Although linear regression (Figure 3c) revealed no clear trend between BC concentration and dATP levels (R^2^ = 0.01), a notable temporal pattern emerged: the increase in dATP over time substantially outpaced that of cATP in all samples (Figure 5). A paired Wilcoxon signed-rank test on the overall change from days 1 to days 7 confirmed that the increase in dATP was significantly greater than that in cATP (stat = 0.0, *p* = 0.002). For instance, in the PM + BC50 sample, cATP increased by only 640 ng mL^−1^ from day 1 (149.6 ng mL^−1^) to day 7 (790.0 ng mL^−1^). In contrast, dATP rose by about 1391 ng mL^−1^ (from 63.1 ng mL^−1^ to 1453.7 ng mL^−1^) during the same period. Notably, this increase was not uniform over time: while dATP increased by 216 ng mL^−1^ between day 1 and day 5, it surged by 1175 ng mL^−1^ between day 5 and day 7, as confirmed by a paired Wilcoxon signed-rank test (stat = 0.0, *p* = 0.002), likely suggesting an accelerated stress response in the later phase of exposure. This divergence suggests that exposure duration may play a more critical role than concentration in triggering physiological responses beyond a certain BC threshold, and prolonged BC exposure could ultimately lead to cell death.

This interpretation is further supported by previous findings that carbon-based nanomaterials (CBNs), including graphene nanoparticles and multi-walled carbon nanotubes, induced cytotoxic effects, with toxicity increasing over prolonged exposure durations [64], and that soot-derived carbon nanoparticles can penetrate bacterial membranes and inhibit bacteria from dividing and multiplying, ultimately causing cell lysis [65]. While such internalization remains a plausible toxicity mechanism, it may not represent the sole pathway through which BC exerts its effects. PA14 has a restrictive outer membrane and lacks endocytic pathways, which likely limits its capacity to internalize particles such as Aquadag [66]. Consequently, interactions with BC particles are likely confined to the cell surface. Nevertheless, even surface-level contact may compromise membrane integrity. Given the crucial role of the cell membrane in ATP synthesis and energy regulation, such damage could interfere with basic cellular functions. For PA14, which relies on membrane potential for the regulation of energy metabolism, these surface-level effects may partially explain the observed elevated dATP levels over time. During the 7-day incubation, the overall decrease in cATP levels over time was firstly due to nutrient depletion, as no additional nutrients were added after the initial setup. On top of this metabolic limitation, exposure to BC seemed to introduce further environmental stress, interfering with the cells’ ability to maintain energy balance. While we did not directly examine biofilm formation, the reducing trend in ATP production, especially the sharp increase in dATP, suggests bacterial membrane damage or cell lysis. This kind of response may reflect a microbial survival strategy, in which cells under long-term stress shift toward protective behaviors, possibly including biofilm-like aggregation [67]. The accumulation of dATP could also signal the release of extracellular ATP, which has been associated with damaged membranes and stress responses [68]. Overall, our results suggest that under nutrient-starved conditions, BC acts as a strong stressor, disrupting microbial energy metabolism and slowing growth—an effect clearly reflected in the shifts in ATP profiles.

### 3.3. Stress Index of Bacterial Growth over Time (BSI)

Environmental stressors such as pH, oxygen concentration, and beneficial or harmful material load are generally considered in the culture conditions [69]. BSI allows for a quantitative assessment of these stressors, making it useful for evaluating their effects on microbial physiology and ecological stability in their growth environment [70,71].

In this experiment, we analyzed the relationship between BC concentration and microbial stress in microbial cultures. Table S1 shows that the mean BSI of the Ctr was 28.4%, while that of the PM + BC0 sample increased to 36.4%, indicating that PM alone functioned as a stressor for microbial communities. In particular, the BSI values in the PM + BC0 sample remained comparable to the control until day 5 but increased markedly by day 7, indicating that PM alone exerts minimal stress effects during early exposure, with stress responses emerging after prolonged exposure. In comparison, the mean BSI across all PM + BC-treated samples was 38.7 ± 14.2%, suggesting that the addition of BC further amplified the physiological stress.

**Figure 6:**
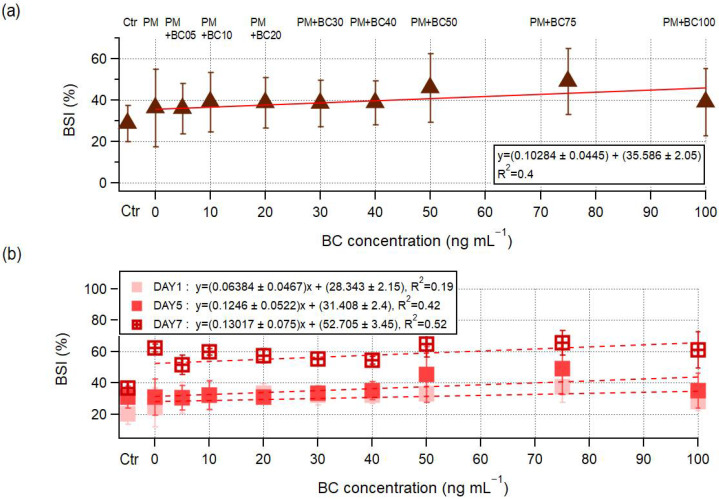
Changes in BSI values in samples with varying BC (Aquadag) concentrations for whole experimental days (**a**) and over time (day 1, day 5, and day 7) (**b**). The red solid line in (**a**) and the red dashed line in (**b**) represent the trend lines of BSI changes.

Figure 6a illustrates the BSI trends across all samples with different BC concentrations over the entire 7-day incubation period, with values ranging from 13.8% to 71.2%. Notably, BSI values remained below 30% in the Ctr, PM + BC0, and PM + BC5 samples, whereas samples containing more than 10 ng mL^−1^ of BC consistently exceeded 30%. A positive trend was observed with increasing BC concentration (slope = 0.102, R^2^ = 0.4), suggesting that higher BC levels moderately contributed to the intensification of the microbial stress response. Meanwhile, temporal changes in BSI values revealed a clear time-dependent increase across all exposure conditions (Figure 6b). BSI values in the bulk samples ranged from 13.8% to 42.8% on day 1, 20.9% to 67.1% on day 5, and 36.7% to 71.2% on day 7. The corresponding mean ± standard deviation values of BSI were 30.4 ± 8.2%, 35.5 ± 10.8%, and 57.0 ± 9.2% on days 1, 5, and 7 (Table S1), indicating that microbial communities experienced progressively greater physiological stress with prolonged exposure duration.

**Figure 7:**
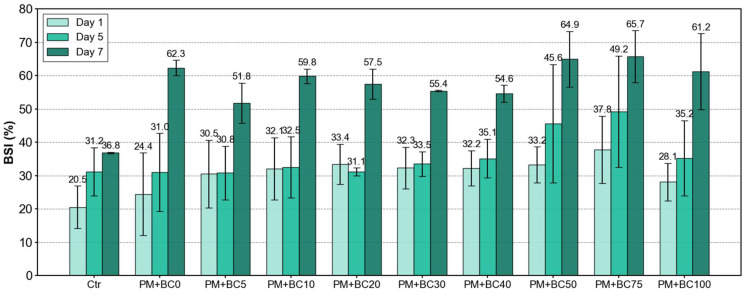
Changes in BSI over time (day 1, day 5, and day 7) across all samples. Black text above each bar indicates the measured average BSI value for each day.

A more detailed examination by day further revealed that BSI values on day 1 showed relatively low variability across BC concentrations (slope = +0.064 ± 0.047, R^2^ = 0.19; Figure 6b), suggesting limited stress in the early phase. In contrast, the relationship became more pronounced from day 5, particularly in samples with BC concentrations ≥ 50 ng·mL^−1^, which exhibited substantially elevated BSI values of 34.4 ± 4.85, 43.3 ± 7.27, and 63.97 ± 2.4 on days 1, 5, and 7, respectively. The BSI slopes increased on day 5 (+0.125 ± 0.052, R^2^ = 0.42), and the highest BSI (72%) was recorded on day 7. Despite continued microbial growth throughout the 7-day incubation, the increasing BSI values suggest that the cells were growing under progressively more stressful conditions. The rise in BSI was most pronounced in samples with BC concentrations ≥ 50 ng mL^−1^ (Figure 7), with the median slope over days 1, 5, and 7 being higher in the ≥50 ng mL^−1^ group (5.0) than in the <50 ng mL^−1^ group (3.3). Elevated cellular damage likely resulted from increasing stress quantified by BSI, leading to a rise in dATP over time (Figure 5b). A Spearman correlation showed a strong positive relationship between BSI and dATP (ρ = 0.822, *p* < 3 × 10^−8^), indicating that higher BSI values were associated with higher dATP levels.

These results suggest that surface interactions between BC and microorganisms lead to a significant negative effect on energy production and maintenance in microbial cells. Ultimately, these results demonstrate that exposure duration, not merely concentration, plays a critical role in the toxicity of BC.

## 4. Conclusions

The effects of BC exposure on microbial growth were examined using a reference material exhibiting similar physical characteristics to urban ambient BC particles. As BC concentration increased from 0 to 100 ng mL^−1^, cATP decreased consistently, while dATP and BSI increased, especially after an extended exposure of seven days. These patterns suggest that BC slows microbial metabolism and induces cellular stress. The most pronounced changes appeared after day 5, indicating that cumulative exposure duration plays a critical role in driving these effects. The time-dependent impacts are likely related to how BC particles physically interact with microbial cell surfaces, which can disrupt essential membrane functions, such as nutrient uptake and membrane potential regulation. These membrane-level interactions may also influence biofilm formation, thus extending the effects of BC beyond basic energy metabolism.

However, Aquadag, used as a BC surrogate in this study, may not fully represent the chemical complexity of BC in ambient environments. In real atmospheric conditions, BC particles are often mixed with various substances generated during combustion processes, such as polycyclic aromatic hydrocarbons (PAHs), heavy metals, and oxidized organics that may further increase the toxicity of the BC-containing particles. In addition, microorganisms used in this study were PA14, a harmful bacterial strain, which may not adequately reflect the responses of non-pathogenic or environmentally relevant microorganisms.

To extend the findings in this study to the context of human health, future research should integrate non-pathogenic or beneficial microorganisms and use BC material derived from real-world environmental or combustion emission sources. The incorporation of these elements would allow a more realistic assessment of BC’s impacts and could provide a stronger scientific basis for policy recommendations on BC emission reduction to protect both environmental integrity and public health.

## Figures and Tables

**Table 1:** Elemental composition and mass fraction with PAH analysis (adopted from [35]).

Elemental Composition	Mass Fraction (%)	
	Measurement *	Measurement Uncertainty
Sb	0.0679	0.0030
Ca	39.5	2.4
Cr	0.307	0.014
Co	0.01996	0.00070
Cu	4.01	0.30
Pb	0.293	0.013
Mg	9.43	0.54
Sn	0.191	0.012
Zn	8.17	0.44
Benz(a)anthracene	0.494	0.044
Benzo(a)pyrene	0.354	0.032
Benzo(b)fluoranthene	1.24	0.12
Benzo(e)pyrene	0.89	0.10
Benzo(ghi)perylene	1.11	0.12
Benzo(j)fluoranthene	0.466	0.046
Benzo(k)fluoranthene	0.420	0.045
Chrysene	1.019	0.097
Indeno(1,2,3-cd)pyrene	0.742	0.087
Triphenylene	0.399	0.039

* The units of measurement for Sb through Zn are g·kg^−1^, and those for benz(a)anthracene through triphenylene are mg·kg^−1^, which were converted to mass fractions (%) in Table 1 for consistency.

**Table 2:** ICP/MS (inductively coupled plasma–mass spectrometry) and WD-XRF (X-ray fluorescence spectroscopy) Measurements, Korea Research Institute of Standard and Science (KRISS).

Analyze	ICP/MS (g·kg^−1^)	WD-XRF (g·kg^−1^)
Na	10.7	13.1
Al	37.0	45.5
K	17.7	15.1
Ti	-	4.1
Mn	1.5	1.2
Fe	99.0	81.2

**Table 3:** Cultivation conditions for PA14 with PM and BC (Aquadag).

Sample ID	Cultured Strain	Concentration (ng mL^−1^)	
		PM	BC
Control	PA14	0	0
PM		5	0
PM + BC5		5	5
PM + BC10		5	10
PM + BC20		5	20
PM + BC30		5	30
PM + BC40		5	40
PM + BC50		5	50
PM + BC75		5	75
PM + BC100		5	100

## Data Availability

Data will be made available on request.

## Supplementary Materials

The following supporting information can be downloaded at: https://www.mdpi.com/article/10.3390/toxics13090719/s1, Table S1. Experimental summary of tATP, dATP, cATP, and BSI of microbial cultivation samples with varying BC(Aquadag) concentrations. The unit for tATP, dATP, cATP, and BSI is ng mL^−1^.
