# Supplementary material for: Inhibitory Effects of Aquadag, a Black Carbon Surrogate, on Microbial Growth via Surface-Mediated Stress: Evidence from Adenosine Triphosphate Assay
Source: Toxics. 2025 Aug 27;13(9):719. doi: 10.3390/toxics13090719 (PMC12473884; doi:10.3390/toxics13090719)
Supplement: Supplementary file 1 [file toxics-13-00719-s001.zip › toxics-3786310-supplementary.pdf]

Inhibitory Effects of Aquadag, a Black Carbon Surrogate, on Microbial Growth via Surface-Mediated Stress: Evidenced from Adenosine Triphosphate Assay

Hwan-Gyu Yoo[a,1,2], Saehee Lim[a,b,2,*], Iseul Cho[b], Haneul Im[b], Euna Lee[b], Siyoung Choi[b], Han-Suk Kim[c,d], Sohee Jeong[b], Younggyun Choi[a,b],

[1] Department of Environmental Engineering, Chungnam National University, Daejeon 34134, Republic of Korea; hwankuy97@gmail.com (H.Y.); youngchoi@cnu.ac.kr (Y.C.)
[2] Department of Environmental IT convergence Engineering, Chungnam National University, Daejeon 34134, Republic of Korea; iseuljo22@gmail.com (I.C.); dlagksmf3020@gmail.com (H.I.); pringles6946@gmail.com (E.L.); swing0128@gmail.com (S.C.); sosohee9@naver.com (S.J.)
[3] Department of Earth and Environmental Sciences, Korea University, Seoul 02841, Republic of Korea; hskim77@dm-ent.co.kr(H-S.K.)
[4] R&D Center, Dong-Myung Enterprise, Seoul 06725, Republic of Korea
* Correspondence: saehee.lim@cnu.ac.kr
† Current address: Industry Coordination Department, Korea Meteorological Institute., Seoul 03735, Republic of Korea
‡ These authors contributed equally to this work.

Table S1. Experimental summary of tATP, dATP, cATP, and BSI of microbial cultivation samples with varying BC(Aquadag) concentrations. The unit for tATP, dATP, cATP, and BSI is ng mL^{-1}.

Sample	Day	tATP		dATP		cATP		BSI	
		mean	sd	mean	sd	mean	sd	mean	sd
Ctr	Day 1	106.2	88.2	15.8	17.3	80.7	64.0	20.5	6.4
	Day 5	667.4	108.9	156.1	29.7	431.3	72.4	31.2	7.2
	Day 7	2944.1	132.1	1824.3	284.0	1119.7	151.9	36.8	0.2
	Day1 - Day 7	1026.1	1215.3	520.6	814.6	471.9	438.4	28.6	8.8
PM+BC0	Day 1	126.2	9.0	25.5	24.6	96.1	21.8	24.4	12.4
	Day 5	733.4	185.9	202.7	16.5	416.6	52.9	31.0	11.7
	Day 7	2462.4	487.6	1528.9	248.0	933.5	239.6	62.3	2.3
	Day1 - Day 7	938.0	1004.1	467.8	666.8	425.6	359.7	36.4	18.7
PM+BC5	Day 1	125.5	20.7	30.1	13.0	85.9	3.3	30.5	10.1
	Day 5	643.7	32.7	216.8	53.7	444.5	49.3	30.8	8.0
	Day 7	1844.4	949.4	983.8	602.9	860.6	346.6	51.8	6.0
	Day1 - Day 7	749.6	802.1	338.6	467.8	414.0	348.4	35.9	12.2
PM+BC10	Day 1	162.8	29.0	39.6	18.3	108.8	3.4	32.1	9.3
	Day 5	675.1	292.8	199.2	57.9	528.1	99.2	32.5	9.1
	Day 7	2234.5	49.0	1335.9	78.7	898.5	29.7	59.8	2.2
	Day1 - Day 7	872.8	887.5	423.5	569.7	463.5	335.7	39.1	14.5
PM+BC20	Day 1	191.0	78.4	54.0	25.9	127.6	53.2	33.4	6.0
	Day 5	725.0	325.2	213.7	106.7	398.2	98.9	31.1	1.2
	Day 7	2143.7	715.1	1249.6	507.7	744.1	4.8	57.5	4.5
	Day1 - Day 7	879.5	880.4	412.8	559.0	383.2	262.5	38.6	12.3
PM+BC30	Day 1	239.8	30.2	63.5	30.1	162.0	22.6	32.3	6.2
	Day 5	630.9	200.7	227.1	108.8	419.7	134.9	33.5	3.7

	Day 7	1934.9	454.1	1071.5	255.8	713.4	13.9	55.4	0.2
	Day1 - Day 7	810.2	745.6	376.8	450.1	396.5	240.6	38.5	11.1
PM+BC40	Day 1	187.9	93.3	77.8	20.7	158.1	22.3	32.2	5.3
	Day 5	754.1	391.2	211.2	73.3	408.5	101.1	35.1	5.8
	Day 7	1780.7	307.6	975.5	211.9	805.2	95.7	54.6	2.5
	Day1 - Day 7	798.4	704.3	352.2	399.8	413.8	276.0	38.9	10.7
PM+BC50	Day 1	225.8	50.0	63.1	40.8	149.6	28.6	33.2	5.4
	Day 5	925.4	451.8	278.9	191.2	404.5	144.0	45.6	17.7
	Day 7	2243.7	55.5	1453.7	153.5	790.0	209.0	64.9	8.4
	Day1 - Day 7	992.6	872.2	491.7	613.9	405.3	287.6	45.8	16.7
PM+BC75	Day 1	187.0	53.5	76.2	25.1	148.0	45.8	37.8	10.1
	Day 5	362.0	98.4	420.9	238.6	285.5	154.0	49.2	16.7
	Day 7	2002.1	558.3	1336.8	523.5	665.2	34.8	65.7	7.8
	Day1 - Day 7	706.4	833.2	520.6	578.7	328.9	233.9	49.0	15.8
PM+BC100	Day 1	194.5	79.0	72.3	30.6	129.5	53.8	28.1	5.6
	Day 5	786.3	296.5	176.5	74.1	292.4	122.0	35.2	11.3
	Day 7	1700.8	552.0	1071.5	531.4	629.3	20.7	61.2	11.4
	Day1 - Day 7	793.0	677.8	361.2	486.5	315.5	219.8	39.0	16.2
All samples	Day 1	174.7	65.7	51.8	30.5	124.6	42.8	30.4	8.2
	Day 5	690.3	264.3	230.3	120.2	402.9	112.9	35.5	10.8
	Day 7	2129.1	516.5	1283.2	382.9	816.0	182.7	57.0	9.2
	Day1 - Day 7	856.7	829.7	426.6	542.8	401.8	293.2	39.0	14.2
